# Impact of Coagulant and Flocculant Addition to an Anaerobic Dynamic Membrane Bioreactor (AnDMBR) Treating Waste-Activated Sludge

**DOI:** 10.3390/membranes7020018

**Published:** 2017-03-23

**Authors:** Guido Kooijman, Wilton Lopes, Zhongbo Zhou, Hongxiao Guo, Merle de Kreuk, Henri Spanjers, Jules van Lier

**Affiliations:** 1 Department of Watermanagement, Faculty of Civil Engineering and Geosciences, Delft University of Technology, Stevinweg 1, 2628BC Delft, The Netherlands; guo_hongxiao@hotmail.com (H.G.); m.k.dekreuk@tudelft.nl (M.d.K.); h.l.f.m.spanjers@tudelft.nl (H.S.); j.b.vanLier@tudelft.nl (J.v.L.); 2 Department of Sanitary and Environmental Engineering, University of Paraiba State, Avenida Juvencio Arruda SN, Bairro Universitario, Campina Grande, Paraíba 58429-500, Brazil; wiltonuepb@gmail.com; 3 School of Environmental Science and Engineering, Sun Yat-sen University, Guangzhou 510275, China; zhouzhongbo-1986@163.com

**Keywords:** AnDMBR, flocculant, membrane fouling reducer, viscosity, anaerobic digestion

## Abstract

In this work, we investigated the effects of flocculation aid (FA) addition to an anaerobic dynamic membrane bioreactor (AnDMBR) (7 L, 35 °C) treating waste-activated sludge (WAS). The experiment consisted of three distinct periods. In period 1 (day 1–86), the reactor was operated as a conventional anaerobic digester with a solids retention time (SRT) and hydraulic retention time (HRT) of 24 days. In period 2 (day 86–303), the HRT was lowered to 18 days with the application of a dynamic membrane while the SRT was kept the same. In period 3 (day 303–386), a cationic FA in combination with FeCl_3_ was added. The additions led to a lower viscosity, which was expected to lead to an increased digestion performance. However, the FAs caused irreversible binding of the substrate, lowering the volatile solids destruction from 32% in period 2 to 24% in period 3. An accumulation of small particulates was observed in the sludge, lowering the average particle size by 50%. These particulates likely caused pore blocking in the cake layer, doubling the trans-membrane pressure. The methanogenic consortia were unaffected. Dosing coagulants and flocculants into an AnDMBR treating sludge leads to a decreased cake layer permeability and decreased sludge degradation.

## 1. Introduction

High-rate anaerobic treatment is a consolidated concept in industry due to the high chemical oxygen demand (COD) removal, energy recovery and low waste sludge production [1]. The success of high rate anaerobic reactors depends on the extent to which hydraulic retention time (HRT) and solids retention time (SRT) can be uncoupled in a system, to keep the slow growing methanogens in the system. Membranes are used for various separation techniques [2], and could therefore also be used for forming an absolute barrier for methanogens in an anaerobic membrane bioreactor (AnMBR). In this way, the HRT and SRT uncoupling in an AnMBR cannot be disturbed by, for example, high total suspended solids or high fats that can compromise the biomass retention in extended granular sludge bed reactors and upflow anaerobic sludge blanket systems. AnMBRs had become a common concept in wastewater treatment over the last decades with many full-scale references. However, despite the fact that membranes can be a cost effective solution [3], still the main drawbacks of AnMBR systems are the energy consumption, membrane fouling and relatively high investment costs [4]. Membrane fouling limits the flux that can be achieved. Cationic flocculant aids (FAs) in an AnMBR treating wastewater are shown to temporarily increase the permeate flux, create a higher permeability of the cake layer, increase the particle size, and allow for a higher effluent quality because of lowered soluble microbial product (SMP) concentrations [5]. In an AnMBR treating sludge instead of wastewater, FAs can have additional benefits. FAs are known lower the viscosity in anaerobic sludge digestion [6]. Earlier studies indicate that a lower viscosity can increase the hydrolysis rate [6,7] which is considered the rate limiting step in anaerobic sewage sludge digestion [8]. Therefore the application of FAs in anaerobic sludge digestion can lead to increased digestion rates by lowering the viscosity [6,9], although there are reports of lower biogas production rates with the addition of FA [10]. A second advantage of FA addition to an AnMBR treating sludge is the increase in maximum SRT that can be applied. With higher SRTs, the solids concentration and thus viscosity in an AnMBR will be higher as well. Since increased viscosity limits the highest attainable SRT because of increasing solids accumulation [11], lowering the viscosity with FAs could increase the maximally attainable SRT in an AnMBR. Besides membrane fouling, another disadvantage mentioned before is the high investment and operational costs. These high costs are mainly caused by the membranes. However, the cake layer that would normally form on the membrane during filtration is dense and compact and will form an excellent barrier for solids [12]. Therefore, despite the advanced developments in membrane technology in the past decades [13], instead of using a membrane, a simple cloth can act as a support for the cake layer as well, lowering the investment and operational costs [14]. A membrane bioreactor equipped with such a cloth instead of a membrane is referred to as anaerobic dynamic membrane bioreactor (AnDMBR). To the authors’ knowledge, the effects of FAs in an AnDMBR treating sludge had not been investigated yet. There is a study that investigates the application FAs in an AnMBR treating sewage sludge [10], but the experiments to investigate the effects of FAs on digestibility of sludge were limited to batch tests and the results were inconclusive. Therefore, in this study we investigate the effects of cationic FA addition in an AnDMBR treating waste-activated sludge (WAS) in batch and continuous experiments. We investigated the methanogenic activity, the extent of sludge degradation, changes in sludge characteristics and trans-membrane pressure (TMP). Conventional anaerobic digestion (AD) at an SRT and HRT of 24 days was compared to an AnDMBR with an HRT of 18 days and an SRT of 24 days, including a period without and a period with FA addition.

## 2. Material and Methods

### 2.1. AnDMBR Setup and Operation

Table 1 gives an overview of the experimental set-up. In period 1, the digester was operated as a daily fed sewage sludge digester without sludge retention. The reactor had a volume of 7 L and was operated at 35 °C. In period 2 and 3, the digester was coupled to a dynamic membrane module with a total filtration area of 0.025 m^2^.

**Figure 1:**
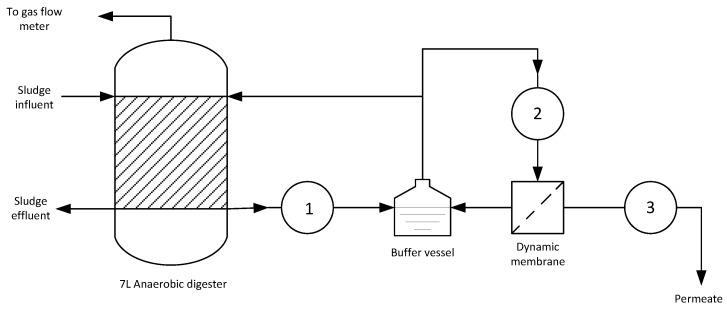
Schematic overview of the AnMBR setup used. The first pump (1) transports the sludge to the buffer vessel from which it is circulated over the membrane by pump 2. The permeate pump (3) creates the pressure difference over the membrane by removing permeate.

The cross-flow velocity over the external dynamic membrane was 0.044 m·s^−1^, which corresponded to a recirculation flow of 240 L·h^−1^. The membrane surface was relatively large for the required liquid extraction from the digester and thus the applied fluxes were very low, reaching only 0.10 L/m^2^·h. No backwash was required. Since in this work we focussed on the biological processes no flux optimisation studies were performed. A mono-filament woven fabric made of polypropylene material (Lampe B.V., Sneek, The Netherlands) was used as support material for the cake layer of the dynamic membrane. Filtration was carried out by using a constant flux strategy set by a peristaltic pump (3 in Figure 1) at the permeate side. The feed and sludge withdrawal was carried out manually, once a day, 6 times per week (not on Sundays). The volume of sludge withdrawal varied and was determined by the sludge concentration and total mass in the reactor and the set SRT (24 days).

The substrate (WAS) was taken from the municipal wastewater treatment plant Harnaschpolder (Den Hoorn, The Netherlands). The WAS had a total solids (TS) concentration between 55 and 65 g·L^−1^. The influent total solids concentration was set to a constant value of 48 g·L^−1^ by diluting the WAS using tap water. The characteristics of the final feed to the digester are shown in Table 2.

### 2.2. FA Selection and Addition

FA was selected by comparing the capillary suction time (CST) and specific resistance to filtration (SRF) of sludge from the AnDMBR treated with 24 cationic flocculants and coagulants. Sludge samples were taken at day 120. An initial screening was carried out by using a CST tests. The six best performing FAs (Table 3), with the shortest CST, were subjected to an SRF test.

The CST and SRF tests were done using a 5 g/kg dosage, which means 5 g of active FA component per kg of TS. From day 267, the best performing FA was dosed to the AnDMBR applying a dosage of 7.5 g/kg. Because no effect was observed at this point in time, from day 303, the Nalco 71305 was replaced with the Caldic (Rotterdam, The Netherlands) cationic FA Calfloc 1502 (10 g/kg) in combination with 40% FeCl_3_ (0.13 mL FeCl_3_ g TS^−1^).

### 2.3. Analytical Methods

Merck Spectroquant kits (Frankfurt, Germany) were used to assess ammonium-N (10–2000 mg-N/L), COD (25–15,000 mg/L) and P concentrations (0.015–5 mg-P/L). Capillary suction time was measured by a Triton Electronics Model 304M CST device (Essex, England, UK). The specific resistance to filtration (SRF) was measured by applying a pressure of 1 bar to a Whatman Grade 1 filter with 100 mL of sludge sample. The permeate volume was measured over time during 2 h. The SRF calculations were done following the procedure of Novak et al. [15]. An Anton-Paar USD200 rheometer with Z2 DIN and TEZ 180 bob (Graz, Austria) was used to measure viscosity. The particle size distribution (PSD) was analysed by a Donner Technologies DIPA-2000 laser scanner (Or Akiva, Israel) with B100 lens, and with 10–2000 µm measuring range. The soluble microbial products of polysaccharide nature (SMP-PS) were measured following the procedure of Ersahin et al. [14]. The soluble microbial products of protein nature (SMP-PN) were measured according to Bradford [16]. The median particle size (D50) was calculated from the volume based PSD. The specific methanogenic activity (SMA) and biomethane potential (BMP) tests were done as previously [6]. The BMP test was done in duplicate, and to each bottle 10 mL antifoam (100× dilution with water) was added. SMA tests were carried out in triplicate. Volatile fatty acid (VFA) concentrations were analyzed using a GC with an FID detector (Agilent 7890A, Santa Clara, CA, USA). Helium was used as carrier gas with a flow rate of 1.8 mL/min. The column used was an Agilent 19091F-112, with injector temperature of 240 °C, 25 m × 320 µm × 0.5 µm, and oven temperature: 80 °C. The remaining parameters were assessed following standard methods of the American Public Health Association (APHA, Washington, USA) [17].

## 3. Results

### 3.1. Performance of the Conventional Sludge Digester and the AnDMBR (Period 1 and 2)

**Figure 2:**
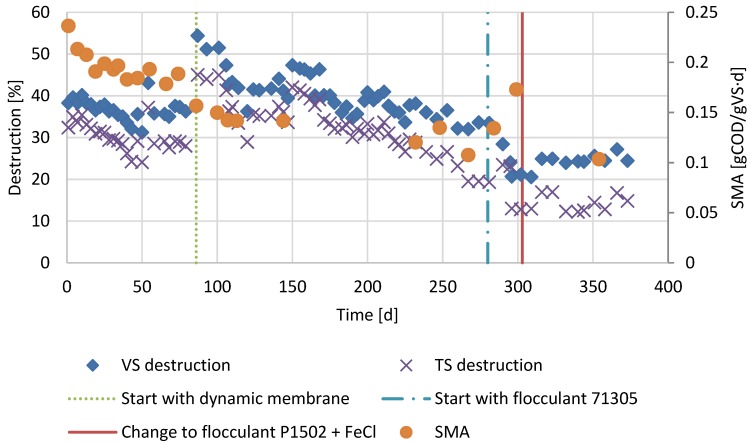
Total solids (TS) and volatile solids (VS) destruction and specific methanogenic activity (SMA) of the reactor operated as conventional anaerobic digester in period 1 (day 1–86), operated as an AnDMBR in period 2 (day 86–303) and operated as an AnDMBR with flocculant addition in period 3 (day 303–386).

In order to study the effect of uncoupling HRT and SRT in sludge digestion, the laboratory scale sludge digester was firstly operated as a conventional digester with an SRT equal to the HRT of 24 days, being fed once per day (period 1). Secondly, in the subsequent period (period 2), the HRT was lowered to 18 days by operating the reactor as an AnDMBR. During the first period, the VS destruction was about 37% (Figure 2). After lowering the HRT to 18 days during period 2, the VS destruction stabilised at about 32%. Also, a slight decrease in SMA could be observed after installing the membrane unit lowering the SMA from 0.19 ± 0.01 gCOD gVS^−1^ d^−1^ to 0.14 ± 0.02 gCOD gVS^−1^ d^−1^.

**Figure 3:**
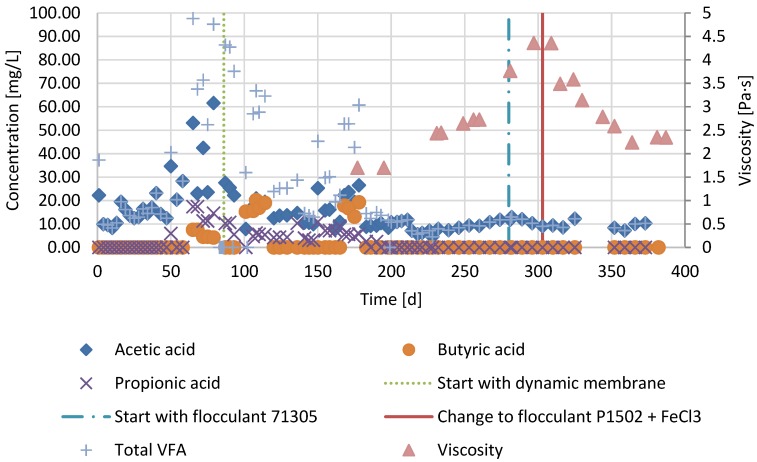
Volatile fatty acid (VFA) concentrations of the reactor and viscosity of the reactor operated as conventional anaerobic digester in period 1 (day 1–86), operated as an AnDMBR in period 2 (day 86–303) and operated as an AnDMBR with flocculant addition in period 3 (day 303–386).

The concentrations of propionate and butyrate remained close to 0 mg/L (Figure 3) during all three periods. Apparently, acetogenic conversions were not rate-limiting in the digester.

### 3.2. FA Selection

**Figure 4:**
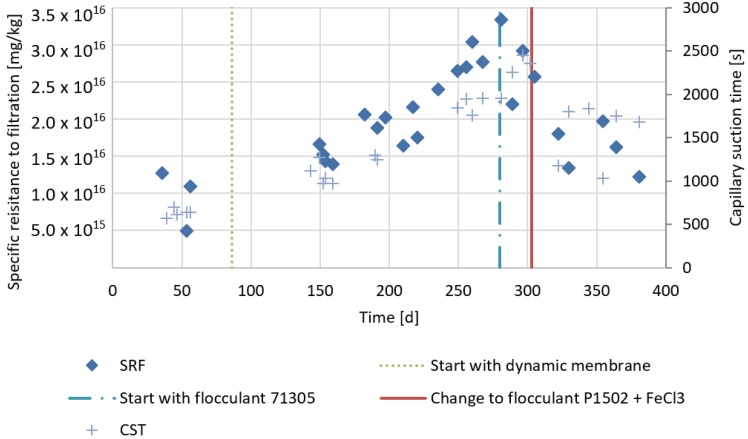
The specific resistance to filtration (SRF) and the capillary suction time (CST) of the effluent sludge of the reactor operated as conventional anaerobic digester in period 1 (day 1–86), operated as an AnDMBR in period 2 (day 86–303) and operated as an AnDMBR with flocculant addition in period 3 (day 303–386).

Twenty-four cationic FAs were tested on the effluent sludge of the AnMBR prior to selection around day 130. The best performing FA in terms of CST and SRF was Nalco 71305. From day 267 onwards, 7.5 g/kg of this FA was dosed. However, FA addition did not result in a visible flocculation in the reactor, the SRF was only shorty affected and CST even increased (Figure 4). Also, repeated CST and SRF tests with an increased dosage of 15 g/kg of Nalco 71305 did not show a clear improvement. Therefore, after one week, Nalco 71305 dosing was stopped. After a new testing phase, the applied FA was changed to a combination of cationic FA Calfloc 1502 (10 g/kg) with FeCl_3_ (0.13 mL FeCl_3_ g TS^−1^). This lowered the CST from ~2000 s to ~500 s. From day 303, a dosage of 10 g kg^−1^ 1502 and 0.13 mL FeCl_3_ g TS^−1^ was applied. Because of the FAs built up in the reactor, the FAs concentrations were lowered to 6.6 g/kg^−1^ and 0.09 mL FeCl_3_ g TS^−1^ from day 330 and to 3.3 g/kg and 0.04 mL FeCl_3_ g TS^−1^ from day 354. The SRF and CST were successfully lowered in the reactor (Figure 4). When dosing the new combination of FAs, foaming problems occurred that were mitigated by adding an antifoam emulsion.

### 3.3. Performance of the AnMBR with FAs dosing (Period 3)

**Figure 5:**
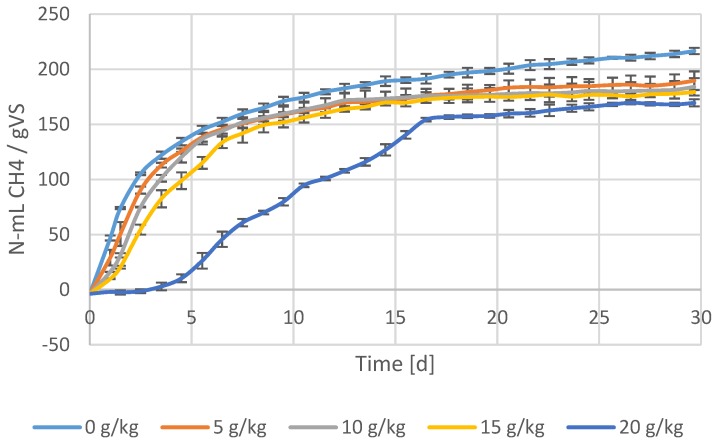
Biomethane potential (BMP) tests of waste activated sludgewith different flocculation aid concentrations. Increased flocculant concentration decreases the BMP values.

During period 3, the viscosity was significantly lowered due to FA addition (Figure 3). In the same period, the VS destruction decreased to about 24% (Figure 2). A BMP test was carried out to examine the possibility of irreversible substrate binding. Results showed that there was already irreversible binding of substrate by FA with dosages as low as 5 g/kg Calfloc 1502 and 0.07 mL FeCl_3_ kg TS^−1^ (Figure 5).

**Figure 6:**
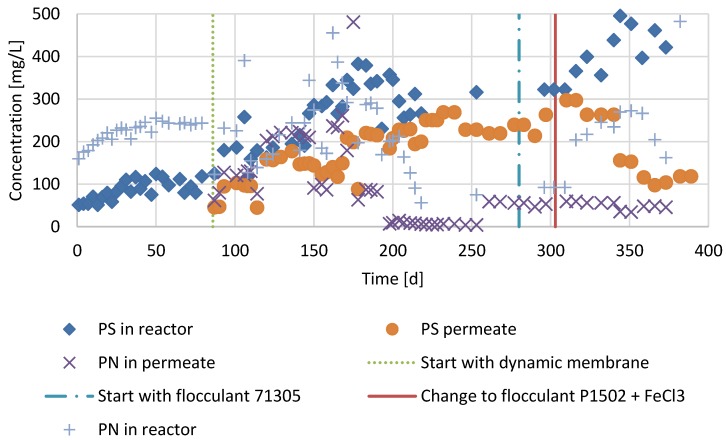
Concentrations of soluble microbial products of polysaccharide nature (SMP-PS) and soluble microbial products of protein nature (SMP-PN) in the supernatant and the permeate of the reactor operated as conventional anaerobic digester in period 1 (day 1–86), operated as an AnDMBR in period 2 (day 86–303) and operated as an AnDMBR with flocculant addition in period 3 (day 303–386).

The addition of FAs in period 3 in the reactor lowered the SRF with about 40% despite the higher TS concentration, reaching to 57 g·L^−1^ in period 3 (Figure 4). Also, the CST decreased in period 3. The average particle size (D50) in period 2 was 58 μm (determined on day 256). Surprisingly, the D50 was reduced after the addition of FAs in period 3 to 32 μm (determined on day 353). The TMP in period 2 was about 150 mbar but it doubled to about 300 mbar in period 3, when FA was added to the digester. The effluent quality increased, as the SMP-PS concentrations in the permeate were lowered in period 3 (Figure 6). At the same time, the SMP-PS concentration in the supernatant of the reactor increased. The permeate SMP-PN concentration remained equal in period 3 compared to period 1. However, the SMP-PN concentrations in the reactor supernatant increased in period 3. 

**Figure 7:**
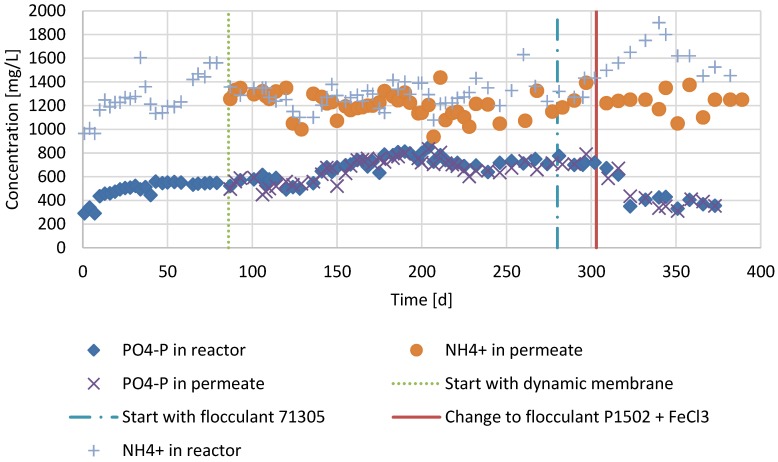
Ortho-phosphate (PO4-P) and ammonium (NH4+) concentrations of the reactor operated as conventional anaerobic digester in period 1 (day 1–86), operated as an AnDMBR in period 2 (day 86–303) and operated as an AnDMBR with flocculant addition in period 3 (day 303–386).

The concentrations of ortho-phosphate (PO4-P) decreased in period 3 (Figure 7). The concentrations were similar for the reactor and permeate. For ammonium (NH4-N), there was an increase in reactor concentration in period 3, while the permeate concentrations remained the same.

## 4. Discussion

### 4.1. Digestion Performance in Period 2

Compared to period 1, the VS destruction in period 2 decreased from 37% to 32% after installing the membrane, while the SRTs in both periods were the same. Lower digester performance was reported earlier, when using an external membrane [18,19,20]. It was postulated that the shear forces caused by pumping the reactor content through the side stream membrane unit caused disruption of the microbial consortia [21]. However, no accumulation of propionic acid or butyric acid was however observed (Figure 3), indicating that syntrophic acetogenic consortia were not notably affected. The treatment performance was also not likely to be affected by free ammonium inhibition as the free ammonium in period 2 was about 30 ± 4 mg/L which is well below the concentration that is found to be inhibiting [22]. Since the VFA concentrations remained low in period 2, the decrease in VS destruction was likely caused by a decreased hydrolysis rate. The higher solids concentration in the reactor compared to period 1 caused a higher viscosity, which likely negatively impacted the hydrolysis rate [6,7].

### 4.2. Digester Performance in Period 3

In period 3, despite the lower viscosity, the VS destruction further lowered to 24%. FAs are considered to be non-toxic to anaerobic consortia [6,9,23]. The acetotrophic methanogens were indeed not notably affected by the FAs, indicated by the similar SMA values in period 2 and 3. In addition, acetogenic conversions and methanogenesis were not the rate limiting step during reactor operation as evidenced by the low VFA concentrations. The treatment performance was also not likely to be affected by free ammonium inhibition, since the free ammonium in period 3 was about 32 ± 2 mg/L. In our previous work, we showed that FAs can irreversibly bind to solids, such that they are not available for bioconversion anymore [6]. Results of the BMP test showed that there was irreversible binding of substrate by FA already with dosages as low as 5 g/kg Calfloc 1502 and 0.07 mL FeCl_3_ kg TS^−1^ (Figure 5). Therefore, with the applied concentrations in the AnMBR, it can be concluded that part of the solids indeed were irreversibly bound, explaining the lower observed VS destruction.

### 4.3. Filtration Performance and Nutrients in Period 2 and 3

The SRF dropped about 40% in period 3 compared to period 2 and the CST dropped slightly. The low drop in CST in period 3, despite the FA addition compared to period 2, may have been caused by the higher TS concentration, which causes higher CST. The lower SRF and CST after FA addition is in agreement with earlier studies in an aerobic MBR [24] and AnMBR [10]. However, the TMP doubled from 150 mbar in period 2 to 300 mbar in period 3. Other studies with AnMBRs, show an increase in filterability due to FA addition [5,10]. The reason for the higher TMP may be an increase of small particles in the reactor in period 3 compared to period 2. Small particles are known to clog the cake in AnMBRs [25]. During AD, colloids are usually rapidly degraded and AD generally causes the average particle size of sewage sludge to increase [26]. That the average particle size in period 3 was lower than in period 2 may be caused by irreversible binding of FA. Cationic FAs irreversibly bind solids [27] and since cationic FAs are known to be partially non-biodegradable [28], the irreversibly bound organic particles can become refractory to biological degradation. Therefore, these refractory particles could accumulate, causing a higher TMP in period 3 compared to period 2. It should be noted that no backwash was applied. From these results it can be concluded that the filtration in terms of TMP did not benefit from the FAs due to the accumulation of small refractory particles that accumulated in the reactor. Typically, the SMP concentrations are lowed by to the addition of FAs [10,29]. However, in period 3, the SMP-PS and SMP-PN concentration increased in the reactor (Figure 6). The increase of SMP in the reactor in period 3 can be explained by the SMP present in the refractory small particles as mentioned above. At the same time SMP concentration in the effluent decreased in period 3, which can be explained by the decreased permeability of the clogged cake in period 3. In Figure 7, it can be observed that the NH_4_^+^ concentration in the reactor increases, while the permeate concentration remains the same in period 3 compared to period 2. This is most likely the result of a measurement bias: cationic flocculants are composed of quaternary ammonium groups, which could be detected as ammonium. Since the flocculant is bound to solids and thus strained by the cake, the permeate did not show the same increase as the reactor content in NH_4_^+^. This hypothesis is supported by the fact that the measured NH_4_^+^ reactor concentration is lowered shortly after lowering the FA dosage on day 330. The PO_4_-P concentration in the reactor and effluent decreased. This was most likely a consequence of the FeCl_3_ dosing.

## 5. Conclusions

An increased viscosity in the reactor, after lowering the HRT to 18 days with a filter cloth, caused a lower VS destruction, most likely due to a lower hydrolysis rate caused by an increased viscosity. Subsequently lowering the viscosity with FAs did not improve the VS destruction. This was explained by an irreversible binding of the substrate. Irreversible binding of organic matter by partially non-biodegradable cationic flocculation aid led to an accumulation of small non-degradable particulates in the reactor. These particulates may have caused a higher TMP caused by pore blocking. The FA concentrations did not notably affect the microbial activity of the system. It can be concluded that FA dosage is not beneficial for WAS treating AnDMBRs.

## Figures and Tables

**Table 1:** Description the three periods where different operational parameters where applied.

Period	Period	Reactor Operation	Substrate	Hydraulic Retention Time (Days)	Solids Retention Time (Days)	Flocculation Aid Addition
Period 1	0–86	Conventional anaerobic digester	WAS	24	24	–
Period 2	86–303	AnDMBR	WAS	18	24	–
Period 3	303–386	AnDMBR	WAS	18	24	Calfloc 1502 + FeCl_3_

**Table 2:** Characteristics of the waste-activated sludge used as feed.

Parameter	Unit	Average Value
Total Solids	g·L^−1^	48.2 ± 1.7
Total Volatile Solids	g·L^−1^	34.9 ± 1.0
Total Suspended Solids	g·L^−1^	45.6 ± 1.6
Volatile Suspended Solid	g·L^−1^	33.9 ± 1.0
Total chemical oxygen demand	g·L^−1^	50.1 ± 3.2
Total Nitrogen	mg·L^−1^	2490 ± 0.515
Total Phosphorus	mg·L^−1^	2435 ± 0.149

**Table 3:** Six best performing flocculants.

Product	Characteristics	Charge
Calfloc L1408	Branched, cationic, emulsion	Medium
Calfloc L111	Branched, cationic, emulsion	Medium
Calfloc L1401 LMW	N.A.	Medium/high
Calfloc P1502	Linear, cationic, powder	High
Calfloc LS1423	Polyamine	Medium
Nalco 71305	Acryl-amide based	High
